# Phenotyping structural abnormalities in mouse embryos using high-resolution episcopic microscopy

**DOI:** 10.1242/dmm.016337

**Published:** 2014-10

**Authors:** Wolfgang J. Weninger, Stefan H. Geyer, Alexandrine Martineau, Antonella Galli, David J. Adams, Robert Wilson, Timothy J. Mohun

**Affiliations:** 1 Centre for Anatomy and Cell Biology & MIC, Medical University of Vienna, 1090 Wien, Austria.; 2 MRC National Institute for Medical Research, London NW7 1AA, UK.; 3 Wellcome Trust Sanger Institute, Cambridge CB10 1SA, UK.

**Keywords:** Phenotype screen, HREM, Imaging, 3D, Episcopic

## Abstract

The arrival of simple and reliable methods for 3D imaging of mouse embryos has opened the possibility of analysing normal and abnormal development in a far more systematic and comprehensive manner than has hitherto been possible. This will not only help to extend our understanding of normal tissue and organ development but, by applying the same approach to embryos from genetically modified mouse lines, such imaging studies could also transform our knowledge of gene function in embryogenesis and the aetiology of developmental disorders. The International Mouse Phenotyping Consortium is coordinating efforts to phenotype single gene knockouts covering the entire mouse genome, including characterising developmental defects for those knockout lines that prove to be embryonic lethal. Here, we present a pilot study of 34 such lines, utilising high-resolution episcopic microscopy (HREM) for comprehensive 2D and 3D imaging of homozygous null embryos and their wild-type littermates. We present a simple phenotyping protocol that has been developed to take advantage of the high-resolution images obtained by HREM and that can be used to score tissue and organ abnormalities in a reliable manner. Using this approach with embryos at embryonic day 14.5, we show the wide range of structural abnormalities that are likely to be detected in such studies and the variability in phenotypes between sibling homozygous null embryos.

## INTRODUCTION

The developmental abnormalities in cellular, tissue or organ structure and function that underlie congenital defects have complex genetic, epigenetic and environmental causes. Furthermore, their incidence and severity is likely to be the result of interactions between multiple contributory factors. Unravelling the aetiology of such defects therefore requires approaches that can both identify the genetic programmes that drive normal embryo development and permit experimental investigation of the impact of non-genetic factors. Although evolutionary conservation of genetic programmes has ensured that much can be learned from a wide range of convenient animal models, such as fruit flies, fish and frogs, a widely used experimental model for developmental disease is now the mouse because it combines the ability to undertake sophisticated genetic manipulation with the ability to study uterine embryo development that more closely resembles that of humans.

Until recently, the majority of advances in understanding genetic controls of murine cellular differentiation and organogenesis have come from studies targeting individual genes or individual genetic pathways for genetic perturbation. However, it has now become feasible to adopt a more systematic approach to such studies. Since 2007, the International Knockout Mouse Consortium (IKMC, www.mousephenotype.org) has been coordinating an international effort to produce knockout lines in which every gene in the mouse genome is individually targeted. This work is now well advanced and attention is now focused on the goal of phenotyping adult mice from the knockout lines, an ambitious task overseen by the International Mouse Phenotyping Consortium (IMPC, www.mousephenotype.org) which is expected to be complete by 2021 (Bradley et al., 2012; Brown and Moore, 2012).

The IKMC knockout programme is also of enormous potential value for understanding the origins of developmental abnormalities, as it provides the opportunity to identify genes whose activity is essential to support normal mouse embryo development in a comprehensive and unbiased manner. Taking advantage of the evolutionary conservation in gene regulatory functions and utilizing the data increasingly available from clinical genetics and genome sequencing studies, it is realistic to expect major advances in our understanding of developmental disease mechanisms from such work (Brown et al., 2006; Cox and Brown, 2003). Furthermore, the identification of genetically modified mouse lines that model aspects of human developmental abnormalities offers the possibility of investigating the complex interactions of environmental factors that can affect disease prevalence and severity.

However, a precondition for such advances is the ability to accurately phenotype the impact of individual gene knockouts on embryonic development. The scale of such phenotyping is considerable; all studies to date indicate that at least one third of the 20,000 gene knockouts will result in embryonic or perinatal lethality and, whilst a proportion will be secondary to placental defects (Adams et al., 2013; Mohun et al., 2013), the overwhelming majority of recessive lethals will result from abnormalities in the development of embryo tissues. Comprehensive screening therefore requires embryos from ~7000 recessive lethal lines to be studied, with multiple embryos studied to take account of variable phenotypic penetrance.

Two observations from previous work help simplify this potentially daunting task. Firstly, whatever the nature of the gene targeted, it is almost invariably the case that embryonic lethal knockout lines show tissue or organ abnormalities that can be identified by morphological criteria, without recourse to molecular or functional analysis. When combined with existing and well-documented gene expression data (e.g. Horsch et al., 2008; Rodriguez-Zas et al., 2008; Seltmann et al., 2005; Zhu et al., 2007), such malformations provide a clue to the developmental function of genes and an entry point for further research. In addition, about half of all affected embryos reach a stage of development (after 14 days of gestation) when normal organogenesis is largely complete and much of the adult-like body plan is established. This therefore provides a useful single developmental window to screen for structural abnormalities, as recognised by the IMPC recommendations for analysis of embryonic lethal gene knockouts (Adams et al., 2013).

**TRANSLATIONAL IMPACT**

**Clinical issue**

This study is embedded in the Deciphering the Mechanisms of Developmental Disorders (DMDD) project. DMDD is part of a global effort to identify mouse strains that have the potential to serve as models for challenging human congenital diseases. By using such models, the molecular and genetic pathways underlying the genesis and progression of developmental diseases can be elucidated. However, a precondition for these studies is the possibility to accurately screen the phenotypes of transgenic mouse lines in order to identify those defects that are associated with embryonic and pre- or perinatal lethality. In order to achieve this, high-resolution imaging of embryos should be combined with more advanced data analysis methods.

**Results**

In this study, the high-resolution episcopic microscopy (HREM) method was used for screening the phenotypes of embryos of 34 mouse strains that produce prenatally lethal homozygous offspring. The authors designed a protocol that allows ergonomic and comprehensive screening and scoring of all relevant phenotype features. They explored the range of abnormalities that can be diagnosed with HREM and found that small to large defects, ranging from the histo-architectural to the macroscopic level, could be precisely identified with the aid of quick virtual resectioning techniques. A total of 58 phenotype abnormalities, which were never described in mouse embryos, could be identified due to the high resolution and quality of HREM data and the systematic phenotyping approach.

**Implications and future directions**

These results demonstrate that HREM combined with a newly designed phenotyping protocol permits the identification of a wide range of previously unidentified structural malformations and tissue abnormalities in prenatally lethal embryos of knockout mice. This indicates that a large number of defects that could be diagnosed with this approach have so far escaped diagnosis using alternative imaging methods. Therefore, HREM should be considered to become the method that is routinely used for screening the phenotype of early mouse foetuses. This is likely to advance our understanding of tissue and organ development and hopefully guide the design of new diagnostic and therapeutic regimes for developmental defects.

Current *in vivo* imaging modalities lack the resolution to assess embryo tissue and organ architecture that is necessary for phenotyping the range of defects associated with embryonic and perinatal lethality. Instead, analysis depends upon the use of *ex vivo* examination of individual embryos. A variety of possible imaging methods are available for such studies [such as optical projection tomography (OPT), micromagnetic resonance imaging (μMRI), microcomputed X-ray tomography (μCT), optical coherence tomography (OCT) and high-resolution episcopic microscopy (HREM)], differing in image resolution and technical complexity, but all sharing the key attribute of providing comprehensive 3D datasets that encompass the entire embryo (Anderson et al., 2013; Metscher, 2009; Schneider and Bhattacharya, 2004; Sharpe, 2003; Tschernig et al., 2013; Weninger et al., 2006; Wong et al., 2012; Zamyadi et al., 2010). However, success in phenotyping will depend not only on the efficacy of the imaging modality. Given the enormous amount of data potentially available for each embryo, it will also require the adoption of analysis methods that are systematic and comprehensive, allowing the comparison of individual datasets and reliable data mining of aggregated results. Crucially, such methods must also be realistic for the scale of the undertaking that is required and tailored to the nature of the data that the imaging modality can yield.

Here, we have investigated the feasibility of using high-resolution episcopic microscopy (HREM), a simple block-face imaging procedure with resin-embedded embryos (Mohun and Weninger, 2012b), to analyse the morphological phenotype of whole-mouse embryos in a consistent and reproducible manner. We have previously shown the efficacy of this imaging method for both qualitative and quantitative analysis of organogenesis in mouse embryos, providing as it does, the highest resolution 3D analysis of embryo morphology currently available (Bamforth et al., 2013; Dunlevy et al., 2010; Geyer and Weninger, 2012; Geyer et al., 2012; Geyer and Weninger, 2013; Mohun and Weninger, 2011; Pieles et al., 2007; Weninger et al., 2009). Using embryos from recessive embryonic and perinatal lethal lines produced as part of the UK contribution to the IKMC knockout programme, we have now assessed the nature, range and probable severity of the structural phenotypes, identified using HREM, and outline a simple screening protocol that enables HREM data to be reviewed in an efficient and timely manner. This protocol provides a way to harness the high resolution of HREM imaging to support an effective and structured method for undertaking systematic and comparative analysis of embryo morphology.

## RESULTS

### A standardised HREM screening protocol

HREM image data resolves tissue and organ morphology at such a high level of detail that establishing a focused, systematic and time-efficient protocol for identifying abnormalities in structure is imperative if HREM is to be used in any screening procedure of comparative analysis. This precluded adopting the use of 3D modelling in our phenotyping procedure as a means of examining all organs and tissues because this would necessitate time-consuming and technically challenging segmentation of datasets. The only use of 3D modelling was therefore in the first step of the protocol, in which a volume-rendered model of the entire embryo was used to define more precisely the embryo developmental stage, using the criteria of external features proposed by Theiler (Theiler, 1989). This was readily achieved using data subsampled to 25% with no appreciable loss in resolution. It also allowed screening for surface abnormalities and major external malformations.

All subsequent analysis was performed using the cubic data set, visualising the captured 2D image set and its two digital, orthogonal plane counterparts (Box 1). Systematic analysis was best achieved by analysing data in section sequence, using axial, sagittal and then coronal planes (supplementary material Figs S1–S3), rather than organ by organ. Using this approach, a standard checklist of features readily discernible in HREM images was established (Box 1; supplementary material Fig. S4). This then formed the basis for systematic phenotyping of each embryo (including those showing multiple and complex abnormalities). Adopting this approach, analysis and documentation of malformations of each embryo took between 1 and 2.5 hours.

**Box 1. Systematic phenotyping protocol for HREM data**

**Step 1: Examine 3D model**

Size

Surface abnormalities, jaws and closure defects

Pinna

Limbs

Theiler stage

**Step 2: Examine axial sections**

Edema?

Brown adipose tissue

Subcutanous haemorrhage?

Brain

*Ventricles*→*choroid plexus*→*tissue architecture*→*internal capsule*

Spinal chord and spinal ganglia

Pituitary gland

Trigeminal ganglion and nerve

Eye, eye muscles and eye stalk and/or optic nerve

Ear

*External acoustic meatus*→*pharyngo tympanic tube*→*cochlea*→*semicircular ducts*→*endolymphatic duct*→*auditory ossicles*

Nasal cavities, nasal septum and vomeronasal organ

Palatine

Tongue and hypoglossal nerve

Oral cavity

Salivary glands

Mandible, styloid process and anlagen of incisor teeth

Hyoid bone

Larynx

Trachea and lungs

Thymus

Thyroid gland

Occipital bone and hypoglossal canal

Topology of head arteries

*Vertebral artery*→*basilar artery*→*labyrinthine artery*→*posterior cerebral artery*→*middle cerebral artery*→*internal carotid artery*

Superior cervical ganglia

Mammary glands

Esophagus and intrathoracic topology of vagal nerve

Stomach

Intestine

*Topology inside embryo*→*topology of umbilical hernia*→*position of caecum*

Bile ducts and gall bladder

Ventral and dorsal pancreas

Spleen

Kidney and proximal ureter

Suprarenal gland

Gonads

Para-aortic bodies

Urinary bladder and distal ureter

Distal mesonephric duct and distal paramesonephric duct

Umbilical and iliac arteries, abdominal aorta, superior mesenteric artery

Osseous pelvis and femur

Lower limb muscles

Abdominal muscles

Scapula, humerus and clavicula

Upper limb muscles

Sternum and ribs

Liver

Great veins of lower body

*Superior mesenteric vein*→*vitelline vein*→*portal vein; umbilical vein*→*portal vein*→*ductus venosus (and valve)*→*hepatic veins*→*inferior vena cava*→*iliac veins*→*hemiazygos and azygos veins*

Thoracal part of azygos vein and great veins of upper body

Lymph sacs

Cardiac ventricles, interventricular septum and atrioventricular junction

Outflow tract and intrathoracic arteries

Atria and interatrial septum

Lung veins

**Step 3: Examine sagittal resections**

Anus

Urethra

Proximal mesonephric duct and proximal paramesonephric duct

Diaphragm and pericardium

Possible reassessment:

*Proximal ureter*, *spleen, pancreas*, *inferior vena cava*, *superior mesenteric vein*

Tibia and fibula

Ulna and radius

Vertebral column and vertebrae (bodies→arcs)

Brain flexures

Innervation of vibrissae

Fila olfactoria

Olfactory bulb

Cranial nerves IX, X, XI

Anterior cerebral artery

Pineal gland

Commissural fibres

Possible reassessment:

*Brain architecture*, *pituitary gland*, *eye muscles*, *larynx*, *edema*

**Step 4: Examine coronal resections**

Position of heart with respect to midline

Semilunar valves

Cranial nerve VII

Cranial nerve VIII

Number of ribs

Possible reassessment:

*Brain ventricles and tissue architecture*, *eyes*, *pituitary gland*, *palatine*, *lymph sacs*, *topology of organs*, *caudate nucleus*

After an initial assessment of developmental stage and external anatomy based on 3D modelling of subsampled HREM data, the protocol outlines the order of organ analysis using the initial axial section plane, followed by digitally derived sagittal and coronal resections. Examination of each organ proceeds from overall topology, through organ morphology to tissue architecture. For some organs, resection data provides a useful opportunity to reassess any possible phenotypes scored earlier in the protocol and these steps are shown in italics.

### Range and penetrance of the abnormalities detected

**Fig. 1.**
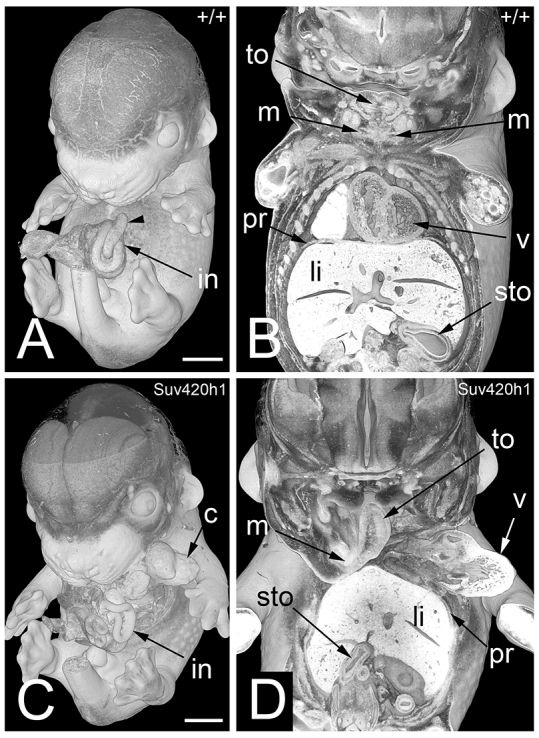
**Multiple defects in a TS21+ mouse embryo (*Suv420h1*^−/−^).** 3D volume rendered models. (A) External aspect of a wild-type (+/+) embryo. Note the intestine (in) forming the umbilical hernia and the caecum (arrowhead). (B) Coronally sectioned 3D model of this embryo. Note the mandibles (m) in the left and right lower jaw, the symmetric appearance of the tongue (to), the diaphragm (pr) cranial to the liver, and the position of the organs. (C) External aspect of a *Suv420h1*^−/−^ embryo. Note the thoracoschisis, the position of the heart (c), and the arrangement of the intestine. (D) Coronally sectioned 3D model of this embryo. Note the following malformations: the left lower jaw is missing. The tongue is thin and fixed only on the right mandible. Cardiac ventricles (v) are outside the body cavity. Stomach (sto) is on the right side. The liver (li) is shifted cranially into the thoracal cavity. Scale bars: 1 mm.

Using the approach outlined above, 34 knockout lines were analysed (Table 1), with three homozygous null embryos compared with wild-type littermates. Of the 102 homozygous null embryos examined, 25 appeared morphologically normal, 27 showed only one or two abnormalities, but approximately half (50) had multiple structural defects (Fig. 1). In total, using the standardised phenotyping protocol, we were able to distinguish 166 different abnormalities, 115 of which could be reasonably described using a set of 109 mouse phenotype (MP) ontology terms (see supplementary material Tables S1, S2). For a further 17 abnormalities, no appropriate MP term was available, necessitating the use of the generic term ‘other defects’ (MP0005395). Thirty-four abnormalities were classified under the broad heading of ‘abnormal blood vessel morphology’ (MP0001614), these largely comprised the absence of segments and/or abnormal topology of arteries (*n*=19) or veins (*n*=15).

**Table 1.**
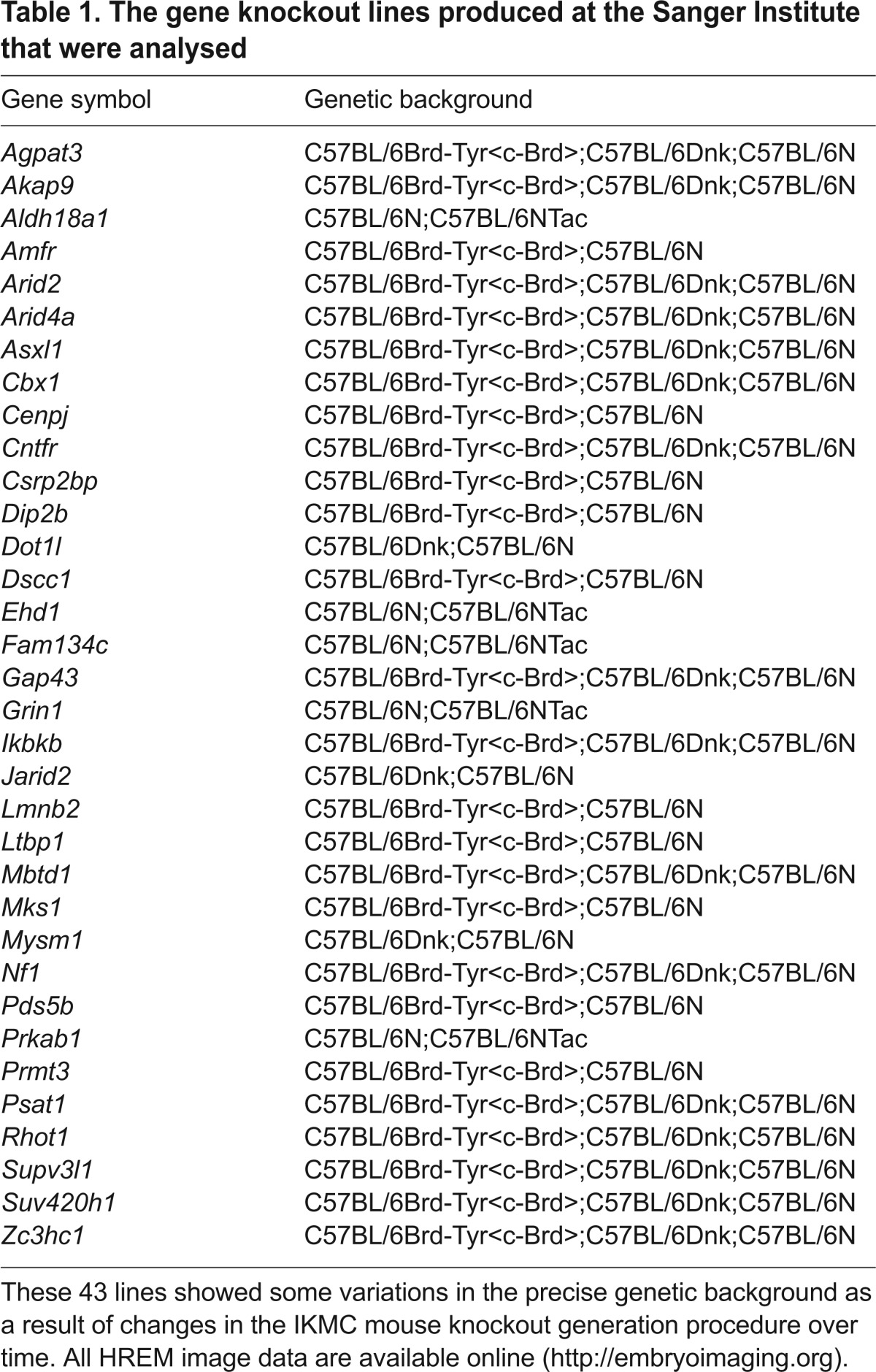
The gene knockout lines produced at the Sanger Institute that were analysed Classifying the severity of abnormalities

A striking result of this pilot study was the variation in phenotype penetrance, with some abnormalities evident in all three homozygotes, whereas the majority were found in only one or two of the three (supplementary material Table S1). For some lines, this resulted from clear variations in severity with which individual homozygotes were affected by the gene ablation, but for many, it appears that variable penetrance of individual abnormalities produced a distinct but overlapping spectrum of lesions in each affected embryo. At embryonic day 14.5 (E14.5), five lines showed no detectable morphological abnormality, indicating either that the lesions responsible for embryonic and/or perinatal lethality are undetectable by HREM imaging, or that they manifest themselves at later stages of embryo development.

### Classifying the severity of abnormalities

**Fig. 2.**
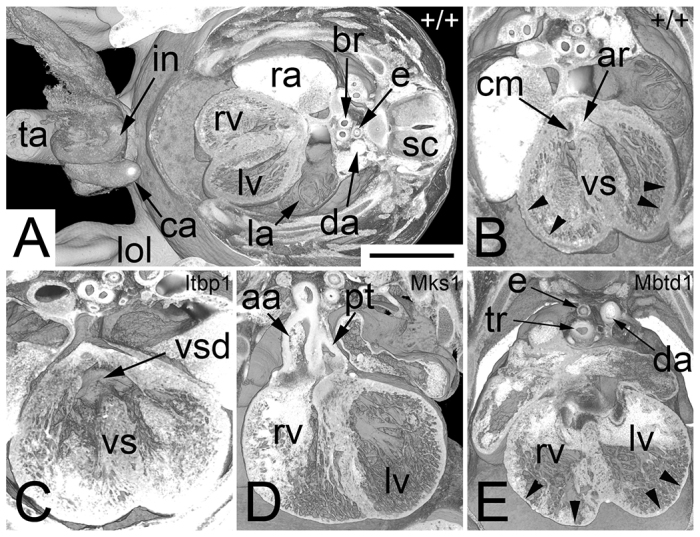
**Abnormalities that cause pre- or perinatal death (Sc1).** Axially sectioned volume rendered 3D models. (A) Wild-type (+/+) embryo. Note the whitish appearance of blood, e.g. in the right atrium (ra). (B) Zoom in on the heart shown in A. Note the blood-filled left ventricular outflow (ar), the cardiac cushion material (cm) that connects to the ventricle septum (vs), and the thickness of the ventricle walls (arrowheads). (C) Perimembranous ventricular septal defect (vsd) in an *Itbp1*^−/−^ embryo. (D) Double outlet right ventricle with transposition of ascending aorta (aa) and pulmonary trunk (pt) in an *Mks1*^−/−^ embryo. (E) Thin compact layer of the myocardium (arrowheads) of the left (lv) and right ventricles (rv) in a *Mbtd1*^−/−^ embryo. br, bronchus; ca, caecum; da, descending aorta; e, esophagus; in, intestine; la, left atrium; lol, lower limb; ra, right atrium; sc, spinal chord; ta, tail; tr, trachea. Scale bar: 1 mm.

**Fig. 3.**
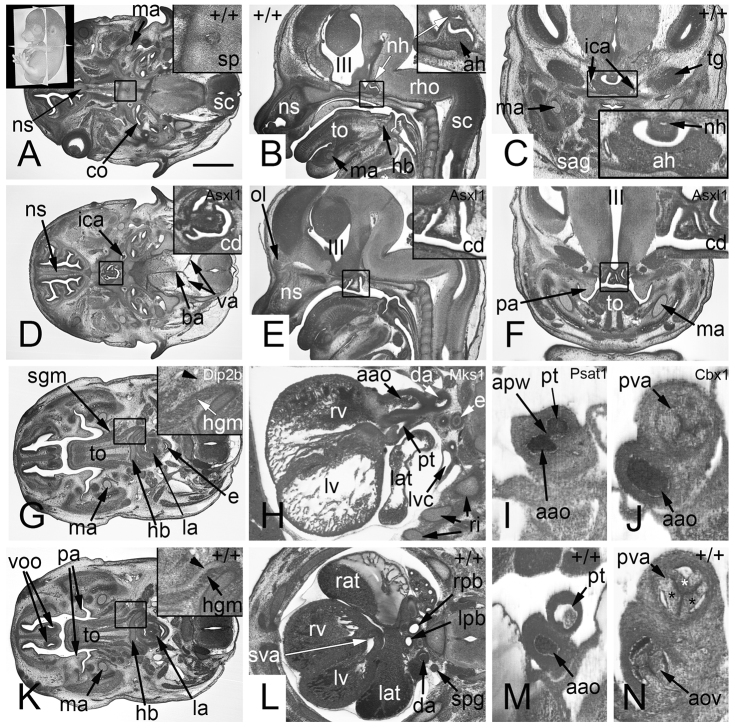
**Abnormalities that cause pre- or perinatal death (Sc1).** 2D orthogonal plane images. (A–C) Control sections (+/+). A corresponds to D, B to E, and C to F. Note the parasellar segment of the internal carotid artery in F. (D–F) Malformation of the adenohypophysis (ah) and absence of the neurohypophysis (nh). Note the visibility of multiple craniopharyngeal ducts (cd) in the inlays (*Asxl1*^−/−^). (D) Axial section (original section plane). Note the narrow basilar (ba) and vertebral (va) arteries. Scale bar: 1 mm. (E) Sagittal resection. Note the normally formed olfactory bulb (ob). (F) Coronal resection. Note the position of the palatine plate (pa) lateral to the tongue (to). (G) Missing tongue (to) innervation in a *Dip2b*^−/−^ embryo. No hypoglossal nerve (arrowhead) exists lateral to the hypoglossus muscle (hgm). Axial section. (H) Double outlet right ventricle (rv), which is associated with transposition of the ascending aorta (aao) and pulmonary trunk (pt) and right-sided descending aorta (da) in an *Mks1*^−/−^ embryo. Axial section. (I) Aortopulmonary window (apw) in a *Psat1*^−/−^ embryo. Coronal resection. (J) Additional cusp of pulmonary valve (pva) in a *Cbx1*^−/−^ embryo. Coronal resection. (K–N) Controls for G to J. (K) Normal position of the hypoglossal nerve (arrow in inlay). Note the vomeronasal organ (voo). (l) Normal position of the subvalvular aorta (sva). Note the left (lpb) and right (rpb) principal bronchii and the paravertebral sympathetic ganglion (spg). (M) Normal topology of ascending aorta and pulmonary trunk. (N) Normal number of cusps (*) of the pulmonary valve. ah, adenohypophysis; aov, aortic valve; apw, aortopulmonary window; ba, basilar arteries; cd, craniopharyngeal ducts; co, cochlea; da, descending aorta; hb, hyoid bone; III, third ventricle; la, larynx; lat, left atrial appendix; lpb, left principal bronchii; lv, left ventricle; ma, mandible; nh, neurohypophysis; ns, nasal septum; ob, olfactory bulb; pa, palatine plate; pt, pulmonary trunk; pva, pulmonary valve; rho, rhombencephalon; rpb, right principal bronchii; sag, salivary gland; sc, spinal chord; sgm, styloglossus muscle; sp, sphenoid bone; spg, paravertebral sympathetic ganglion; sva, subvalvular aorta; tg, trigeminal ganglion.

In order to rank the abnormalities in terms of severity, we split them into five categories (Sc1–Sc5; supplementary material Table S2). The first severity category (Sc1) encompassed 30 abnormalities that individually are highly likely to cause pre- or perinatal death. Examples include severe heart defects, absence of the jaws, absence of the corticospinal tracts, absence of the hypoglossal nerves, intramural bleeding in the aortic wall and absence of the tongue muscles (Figs 2, 3).

**Fig. 4.**
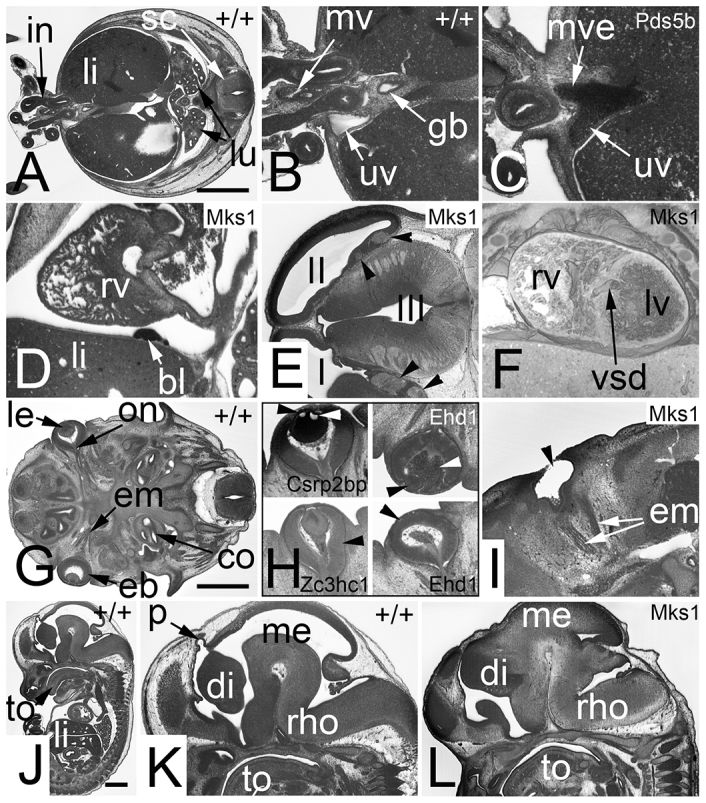
**Severe and dangerous defects (Sc2, Sc3).** Potential pre- or perinatal life-threatening defects (Sc2 in A–F). Abnormalities that reduce life span (Sc3 in G–L). (A) Axial section through a wild-type (+/+) embryo. Note the intestine (in) forming the umbilical hernia and the liver (li). (B) Zoom into the junction of umbilical hernia and body. Note the umbilical vein (uv) right to the intestine and the mesenterial vessels (mv) between the intestinal segments leaving and entering the body. (C) A *Pds5b*^−/−^ embryo in which the vitelline vein (mve) connects with the umbilical vein. (D) Hemangioma-like lesion (bl) in the pericardium of an *Mks1*^−/−^ embryo. Sagittal resection. (E) Fibroma (arrowheads) in the telencephalon of an *Mks1*^−/−^ embryo. Axial section. (F) Muscular ventricular septal defect (vsd) in an *Mks1*^−/−^ embryo. Coronally sectioned 3D model. (G) Axial section through a wild-type embryo. Note the form and position of the eyeball (eb), lens (le), optic nerve (on) and eye muscles (em). (H) Various eye defects are indicated with arrowheads. Vacuolated lens at top (*Csrp2bp*^−/−^) left. Displaced lens (black arrowhead) and retro-lental bleeding (white arrowhead) are visible at the top right (*Ehd1*^−/−^). A malformed eye ball (abnormal optic cup) is shown at the bottom left (*Zc3hc1*^−/−^). An absent lens (vesicle) is marked at the bottom right (*Ehd1*^−/−^). (I) A missing eye ball (anophthalmia) in an *Mks1*^−/−^ embryo. Note that the eye muscles do exist. (J) Sagittal resection through a wild-type embryo. (K) Zoom in of the head region of embryo displayed in J. Note the pineal gland vesicle (p). (l) Missing pineal gland vesicle in an *Mks1*^−/−^ embryo. Note that also the rest of the diencephalon (di) is malformed. co, cochlea; gb, gall bladder; I, first brain ventricle; II, second brain ventricle; III, third brain ventricle; in, intestine; lu, lung; lv, left cardiac ventricle; me, mesencephalon; rho, rhombencephalon; rv, right cardiac ventricle; sc, spinal chord; to, tongue. Scale bars: 1 mm.

The second category (Sc2) contained a further 18 abnormalities that could well result in pre- or perinatal lethality, especially if combined with other defects. Examples include the presence of multiple fibroma in the brain, the draining of the mesenteric vein into the umbilical vein, simple perimembranous ventricular septal defects, blood filled cysts in the liver and the unilateral absence of the internal carotid artery (Fig. 4).

The third category (Sc3) comprised 32 abnormalities that individually are unlikely to cause pre- or perinatal death, but that are likely to cause a diminished extra-uterine life span. Examples are defects of the optic cup, the absence of eye muscles, simple muscular ventricular septal defects, defects of the perirectal tissues and the absence of the neurohypophysis (Fig. 4).

**Fig. 5:**
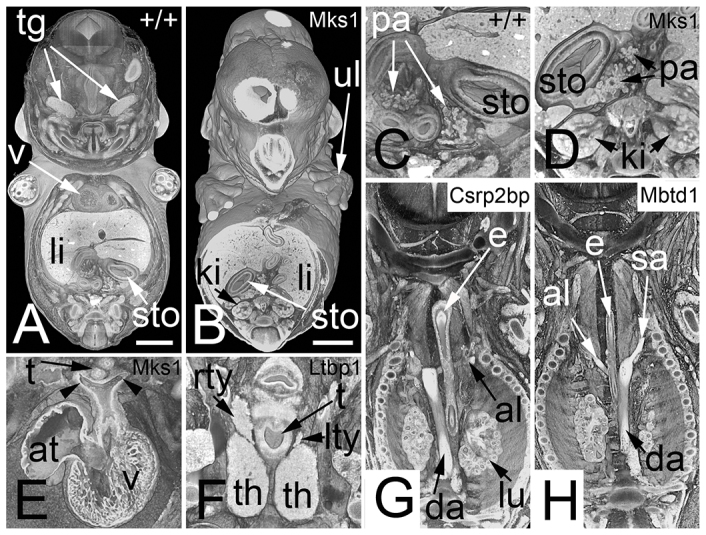
**Abnormalities that are combined with or are indicators for life-threatening defects (Sc4).** (A) Coronally sectioned, volume rendered, 3D model of a wild-type (+/+) embryo. Note the position of the stomach (sto), highlighted in C. Note the positions of the ventral and dorsal pancreas (pa). (B) *Mks1*^−/−^ embryo with a right-sided stomach, highlighted in D. Note that there is only right-sided pancreas tissue. (E) Symmetric left- and right-sided (arrowheads) aortic arch, forming a vascular ring around the esophagus (*Msk1*^−/−^). Note the abnormal position of the atrium (at). (F) Hypoplasia of the left lobe (lty) of the thyroid gland in a *Ltbp1*^−/−^ embryo. (G) Retro-esophageal left subclavian artery (al) in a *Csrp2bp*^−/−^ embryo. Note the inverse position of the descending aorta (da). (H) Retro-esophageal right subclavian artery in a *Mbtd1*^−/−^ embryo. e, esophagus; ki, kidney; li, liver; rty, right thyroid lobe; sa, subclavian artery; t, trachea; tg, trigeminal ganglion; th, thymus; ul, upper limb; v, ventricle. Scale bars: 1 mm.

Thirty of the abnormalities detected (category Sc4) were defects that are not lethal per se, but that were often combined with heart defects or were strong indicators that littermates of the affected embryo were likely to show severe life-threatening defects. Examples are right- or left-sided retro-esophageal subclavian artery, absence of lobes of the thyroid gland, subcutaneous edema and dual inferior vena cava (Fig. 5).

**Fig. 6:**
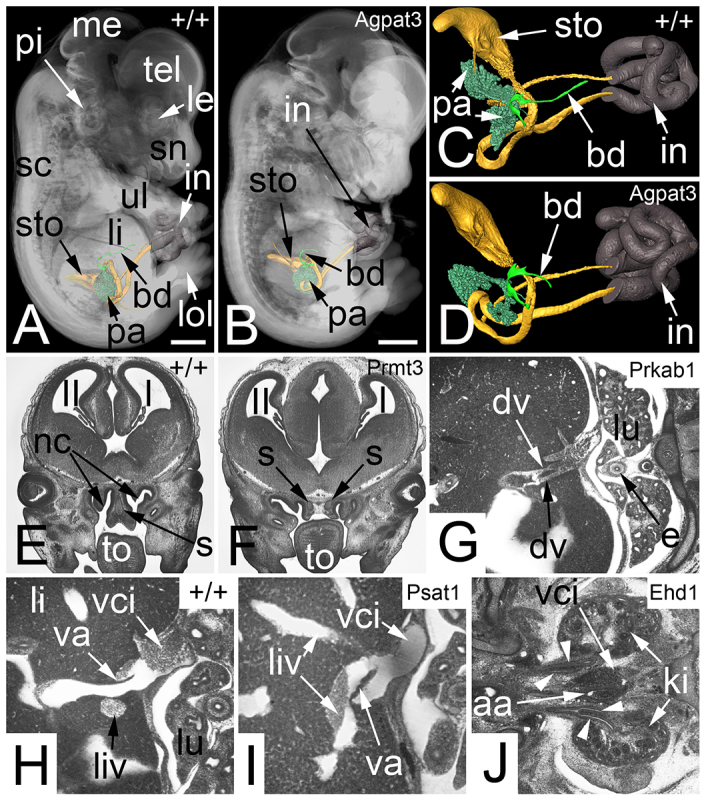
**Abnormalities that are not obvious indicators for severe defects (Sc5).** (A) Transparent volume rendered 3D model of a wild-type (+/+) embryo combined with surface rendered 3D models of the stomach (sto), pancreas (pa), cystic duct (bd) and intestine (in). (B) *Agpat3*^−/−^ embryo of the same developmental stage and displayed in the same modality as A. Note the shortness of the cystic duct. (C) Zoom in of A. The cavity of the stomach and the lumen of the intestine (in) are yellow, the wall of the intestine is dark brown and indicates the umbilical hernia. (D) Zoom in of B. (E) Coronal resection through the head of a wild-type embryo. Note the tongue (to) and the nasal septum (s) between the nasal cavities (nc). (F) *Prmt3*^−/−^ embryo with two nasal septa and a connective tissue space in between. (G) Dual ductus venosus (dv) in an axial section of a *Prkab1*^−/−^ embryo. (H) Axial section through a wild-type embryo. Note the position of the valve of the ductus venosus (va). (I) Axial section through a *Psat1*^−/−^ embryo. Note the abnormal position of the valve of the ductus venosus at the connection with a left-sided liver vein (liv). (J) Abnormal ureters in an *Ehd1*^−/−^ embryo. Note two ureters on the right side and a bifid ureter on the left side (arrowheads). aa, abdominal aorta; dv, ductus venosus; e, esophagus; I, first ventricle; II, second ventricle; ki, kidney; le, lens; li, liver; lol, lower limb; lu, lung; me, mesencephalon; pi, pinna; sc, spinal chord; sn, snout; tel, telencephalon; ul, upper limb; va, valve of ductus venosus; vci, vena cava inferior. Scale bars: 1 mm.

The last category (Sc5) included 54 abnormalities for which the impact on embryo development or survival was difficult to assess. Examples include abdominal situs ambiguous, polydactyly, herniated liver tissue, shortening of the gall ducts, rib abnormalities and duplication of ductus venosus and bifid ureter (Fig. 6).

### Comparison with previously reported phenotypes

Several of the lines we have studied ablate genes that have previously been targeted in other mouse lines and for which some phenotyping data are available from the Mouse Genome Informatics (MGI) database. Direct comparisons are difficult as much of these published data are derived from studies of neonates or adults and encompasses behavioural, biochemical and neurological studies that have no counterpart in our screen.

In the case of *Psd5b*, previous analysis of homozygous null embryos has been undertaken at multiple stages from E12.5 through to term. Using a combination of histological analysis, immunocytochemistry and *in situ* hybridisation, Zhang and colleagues detected growth retardation, disproportional heads and limbs, facial dysmorphism, various skeleton anomalies, anomalies of the heart, abnormalities of the superior cervical ganglia, anomalies of the enteric nervous system of the large intestine and reduced numbers of germ cells in the gonads (Zhang et al., 2007). Likewise, in our study, we have detected a similar spectrum of abnormalities, with developmental delay and/or growth retardation, a variety heart defects, skeletal abnormalities and abnormalities of the superior cervical ganglia, which was identifiable in at least one of the three homozygotes imaged (supplementary material Table S1).

HREM imaging proved inappropriate for detecting abnormalities in germ cells or the enteric nervous system; however, it was able to detect many additional defects, which were unreported in the earlier study. These included severe abnormalities of the head arteries, the suprarenal gland and derivatives of the vitelline veins, all of which constituted serious lesions that could impair embryo viability. Interestingly, these included the absence of the hypoglossal nerve in one of the embryos. Other minor phenotypes evident in HREM included enlarged lymph sacs, eye lens defects, abnormalities of the liver and an abnormal topology of the intestine.

## DISCUSSION

By combining automated data acquisition with very high resolution, HREM provides a powerful way to examine embryo morphology in both 2D image stacks and 3D models. However, the extent of the structural detail it can yield raises important questions about how best to score embryo and organ structure in a manner that is suitable for comparative analysis or screening studies.

One emerging possibility is the use of computer algorithms to compare embryos, an approach that can, in principle, be extended to HREM data (unpublished data), but which is currently rather limited in the range of morphological structures that can be defined in an automated manner. The alternative is to use expert review of image data. Here, the challenge is to devise a procedure that is sufficiently structured to combine comprehensive phenotyping with standardised assessment, and which is sufficiently rapid in order to be useful in embryo screening protocols. In this study, we have described such an approach and present the results of a pilot study examining the morphological abnormalities in embryos at E14.5 from 34 recessive lethal knockout lines of the IKMC-coordinated gene-knockout screen. The pilot study not only provided a rigorous test of the phenotyping procedure but also offered an indication of the range, severity and penetrance of individual abnormalities that can be readily identified from HREM data.

With modern 3D imaging techniques, such as HREM, accurate and reproducible phenotyping is not constrained by limitations in the available image data. Each embryo data set in our study comprised 3000–4000 serial images, with an isotropic resolution for the entire 3D data volume of only 3 μm. The range of tissues and structures that can be distinguished by using HREM enables abnormalities ranging from gross organ malformations to the loss of cranial nerves, white matter defects and the inappropriate branching or location of blood vessels to be detected. Nor, with modern computing resources, is there any technical difficulty in visualising and reviewing such large data sets (~20–30 gigabytes), at least in the form of 2D image stacks. Rather, the scale and detail of the image data poses the challenge of devising a simple procedure that examines a broad range of organs and tissues in an easy and sequential manner.

Having initially used 3D modelling to examine gross external morphology, identify obvious malformations and assess developmental stage, our protocol only uses the captured dataset (axial or transverse) and its two computer-generated orthogonal counterparts, rather than the more demanding 3D modelling or creation of virtual, oblique 2D section views. Each orthogonal view proved to be useful for different aspects of morphology, and the checklist of features to be scored (along with associated MP terms) was designed to suit progressive viewing of each section series in consecutive order. In choosing the features to score through the protocol, we attempted to reconcile the wide-ranging nature of the defects that were detected by HREM imaging (ranging, for example, from gross organ malformations to loss of individual nerves or abnormalities in blood vessel branching) with the need for a procedure that is suitably straightforward and rapid for use in the systematic screening of recessive lethal lines. Using the current screening protocol, it is feasible for a single anatomist to analyse an entire knockout line in a day, including documenting scored phenotypes with formal descriptions [e.g. using MP or mouse anatomical entity (MA) ontologies] (Eppig et al., 2012; Hayamizu et al., 2005; Smith and Eppig, 2009; Smith and Eppig, 2012) and simple image annotation. Perhaps counter-intuitively, unless detailed morphometry is envisaged, the bottleneck facing embryonic lethal screens is unlikely to be the phenotyping itself, but rather the rate production of mutant lines and their embryos for analysis.

To describe the abnormalities in a standardised manner, we adopted the internationally acknowledged MP ontology system (http://www.informatics.jax.org/searches/MP_form.shtml). For most of the abnormalities, this ontology provided terms to describe the abnormalities with sufficient accuracy; however, significant exceptions were the majority of blood vessel abnormalities, for which no appropriate MP terms were available. This was because many of the vessel abnormalities that we detected were topological rather than structural in nature, for example, vertebral arteries that did not enter the spinal canal between the atlas and occipital bone. Similarly, for embryos in which no artery existed in the foramina of the transverse processus of the atlas or other cervical vertebrae, the closest appropriate MP term was ‘abnormal vertebral artery morphology’ (MP 0011513). However, this does not in our opinion adequately describe a defect resulting from agenesis of a normal vascular segment. Any phenotyping screen will therefore need to generate additional agreed terms to meaningfully describe and annotate the full range of abnormalities detected.

Furthermore, HREM data only permits the identification of abnormal tissue arrangements and is not capable of providing information about abnormal cell morphology or abnormal composition of the non-fibrous components of the extracellular matrix. Thus, it cannot reliably establish the true nature of tissues. For example, the abnormal structures inside the brain tissue shown in Fig. 4 could in principle be caused by the proliferation of various tissues. According to the gross appearance, we have classified these defects as neurofibroma, but confirmation of this would require immunohistological examination.

Our phenotyping protocol is based on qualitative judgments of morphology and cannot detect with any reliability any changes in overall organ volume or shape that remain subtle in 2D images. Such quantitative measurements are of course possible from 3D data sets and could add a further and truly objective aspect to embryo phenotyping. Indeed, such an approach, initially pioneered for comparison of MRI data has now been adapted to the higher resolution data sets that have been obtained using μCT. Although the results of such studies are encouraging, they also demonstrate the challenges facing the development of automated phenotyping. Automated comparison of mutant 3D data sets with statistically averaged reference data requires manual identification and segmentation of individual organs, regions or tissues (Cleary et al., 2011; Dorr et al., 2008; Kovačević et al., 2005; Wong et al., 2012), a procedure which has only so far proved effective with a subset of embryo organs. The alternative is comparison that is independent of organ identification, a procedure which can yield ‘heat maps’ of voxel pattern variation that can help identify regions of possible structural variation (Wong et al., 2014). Such approaches could in principle be applied to HREM data, taking advantage of the greatly increased resolution of individual structures to expand the range of features that can be included in the automated comparisons. However, it should be noted that because normal organs and tissues show differing degrees of variability in dimensions (in part, presumably, owing to a degree of heterochrony in development), the sensitivity and confidence with which size changes can be detected by automated procedures varies considerably, potentially necessitating the study of substantially larger mutant embryo cohorts and a consequent increase in screening costs. Furthermore, as previously noted (Anderson et al., 2013; Wong et al., 2012; Zamyadi et al., 2010), a number of organs are sufficiently variable in morphology that they are unsuited for inclusion in automated detection.

A striking result of this study was the extent to which individual recessive lethal gene knockouts resulted in pleiotropic effects. Our finding that half of the mutant embryos studied had multiple morphological defects could have several explanations. One limitation inherent in screens of this nature is the adoption of a single time point (in this case E14.5) at which to compare mutant and wild-type embryos, with the result that the malformations detected could be both primary results of the gene knockout and secondary abnormalities resulting from perturbed development. Multiple phenotypes could also indicate several distinct roles for the targeted gene during tissue differentiation or morphogenesis. Resolving these possibilities ultimately requires a detailed study of the targeted gene expression and function; however, some clues might be provided by the nature of the malformations themselves if they are consistent with a single developmental origin (e.g. neural crest derivatives) or reminiscent of an established congenital syndrome. Gene knockouts might also contribute to a lethal embryo phenotype through direct impairment of placental function or development and, for this reason, any comprehensive screen to analyse recessive lethals should also include systematic assessment of placental structure (Adams et al., 2013). As this was not included in our pilot study, we cannot assess the extent to which placental abnormalities contributed to the range of embryo defects observed. In principle, HREM could provide a way of imaging 3D placental structure; however, the prevalence of blood in this tissue limits the usefulness of this approach and 2D assessment using standard histology is likely to be more effective.

One of the challenges highlighted by our pilot study is the comparison of mutant and wild-type data sets. Effective phenotyping needs to take into account the effects of normal heterochrony in embryo development such that, for example, amongst wild-type embryos, one might show osseous structures that are more developed than its littermate, whereas its nervous system might be less developed. This complexity is compounded by variability in the precise developmental stage of harvested embryos. We used E14.5 embryos in our study and found a wide variation in the stages, as assessed by the more detailed criteria of Theiler, embryos ranging in fact from stages 21 to 23. Some apparent abnormalities (for example minor ventricular septal defects) could therefore result from developmental heterochrony or indeed from developmental delay, resulting from the gene deletion itself. Although distinguishing a genuine malformation from developmental delay or natural developmental variation will remain a subjective judgment, the key to its success will be to use high resolution imaging techniques to generate detailed maps of organ development over the transition from the embryonic to the fetal period, based on sufficient numbers to reveal the extent of normal variation (see for example http://embryoimaging.org) or the prevalence and penetrance of particular abnormalities that are associated with a particular inbred mouse background. Our pilot study was performed in the absence of such a comprehensive compendium of data and was further complicated by the variations in genetic background that have occurred as the IKMC knockout programme has evolved. A second challenge for maximising the effectiveness of phenotyping is to establish the most effective methods for achieving blood removal from embryos. In most imaging methods, including HREM, blood appears as dense material, obscuring adjacent structures, especially within the heart and great vessels. Because 25–30% of all knockout embryos are expected to show a cardiac phenotype at E14.5–15.5 (Adams et al., 2013), optimising exsanguination procedures will have a major impact on successful cardiac phenotyping.

A number of imaging modalities have previously been used to screen mouse embryos for morphological defects, most notably μMRI (Bamforth et al., 2012; Berrios-Otero et al., 2009; Cleary et al., 2011; Cleary et al., 2009; Schneider et al., 2004; Turnbull and Mori, 2007) and μCT (Degenhardt et al., 2010; Johnson et al., 2006; Wong et al., 2012). In testing HREM as a screening procedure, we attempted to identify the extent to which the enhanced imaging resolution translated into improved phenotyping. As an approximation, we have categorised the abnormalities that we detected into three groups, macroscopic (m), intermediate (i) and histologic (h), according to the level of resolution judged as being necessary for their detection (supplementary material Table S2). Interestingly, approximately one third (58 of 160) of all the abnormalities identified are likely to be detected only with high-resolution (HREM or histology) techniques. These include a large number of life-threatening defects. An additional 45 abnormalities might, in principle, be detectable in some form by using lower resolution methods (μMRI, OPT or μCT), but certain identification would again require confirmation by a high-resolution technique. The adoption of high-resolution screening therefore directly contributed to reproducible diagnosis of nearly two thirds of all the abnormalities that were identified in our screen.

## MATERIALS AND METHODS

### Embryos

Recessive embryonic lethal lines were produced as part of the Mouse Genetics Project at the Wellcome Trust Sanger Institute (https://www.sanger.ac.uk/mouseportal/). Lines were identified as recessive embryonic or perinatal lethal by the absence of any homozygote mutants amongst a minimum of 28 progeny, genotyped 2 weeks after birth (14 days postpartum). For each of 34 knockout lines, three homozygous null embryos and a single wild-type littermate control were analysed. These came from a variety of genetic backgrounds, based upon C57BL/6 (see Table 1), and were identified by yolk sac genotyping. Although small, such numbers afford an 80% confidence of detecting phenotypes that are only 50% penetrant and form the basis of the UK screening programme (Adams et al., 2013; Mohun et al., 2013). Embryos were harvested at approximately E14.5 and immediately transferred to Bouin’s fixative for a minimum of 24 hours. After extensive washing in phosphate-buffered saline and dehydration through a methanol series (10–90% in 10% increments followed by 95% and 100%; a minimum of 1 hour each step), all samples were embedded in JB4 methacrylate resin (Polysciences) containing eosin and Acridine Orange, as previously described (Mohun and Weninger, 2012a). A 1–2 mm cushion of polymerised resin was used to ensure samples remained entirely embedded within the resin without contacting the block surface. Embryos were oriented in the embedding process as consistently as possible, such that transverse sections were obtained from crown to rump. Blocks were left to polymerise overnight at room temperature and then baked at 90°C for a minimum of 48 hours to ensure a uniform, hard texture prior to sectioning. Samples were subsequently cooled and stored at 4°C before imaging.

### Imaging

Embryos were analysed by HREM, using 3-μm sections, green fluorescent protein filters and a Hamamatsu Orca HR CCD camera to obtain the high-resolution images, as previously described (Mohun and Weninger, 2012b). Data sets comprised ~3000–4000 8-bit images (~35 GB total) and were normalised for any fluctuations in exposure. Pixel grey levels were adjusted using a Photoshop 6 (Adobe) macro to optimise tissue contrast, and each dataset was subsampled to obtain cubic voxels.

All HREM image data for the 34 mutant lines analysed is publically available at full resolution (http://embryoimaging.org) along with summary movies of all three orthogonal section plane stacks.

### Data analysis

Cubic (3×3×3 μm^3^) voxel datasets were analysed using Amira 5.4 software (Visage Imaging) using a 64-bit PC workstation (Microsoft Windows 7; minimum of 72 GB RAM). For the routine morphology screen of the pilot study, embryo morphology was assessed in a defined sequence using both 3D volume-rendered models and 2D images from each orthogonal section plane, using a standard protocol (Box 1 and below).

For illustration, volume rendering was combined with arbitrary section plane erosion in Amira to obtain the embryo models shown in the figures. In some cases, individual organs were also digitally segmented from image stacks in Amira and used to generate surface-rendered pseudo-coloured 3D organ models, which were superimposed in an appropriate location upon semi-transparent volume rendering of the whole embryo.

## Supplementary Material

Supplementary Material
