# Supplementary material for: Phenotyping structural abnormalities in mouse embryos using high-resolution episcopic microscopy
Source: Dis Model Mech. 2014 Oct;7(10):1143–52. doi: 10.1242/dmm.016337 (PMC4174525; doi:10.1242/dmm.016337)
Supplement: Supplementary Material [file supp_7_10_1143__index.html]

Supplementary Material 

# Phenotyping structural abnormalities in mouse embryos using high-resolution episcopic microscopy

## DMM016337 Supplementary Material

**Files in this Data Supplement:**

- **Supplementary Material**
