# Supplementary material for: Phenotyping structural abnormalities in mouse embryos using high-resolution episcopic microscopy
Source: Dis Model Mech. 2014 Oct;7(10):1143–52. doi: 10.1242/dmm.016337 (PMC4174525; doi:10.1242/dmm.016337)
Supplement: Supplementary Material [file supp_7.10.1143_DMM016337.pdf]

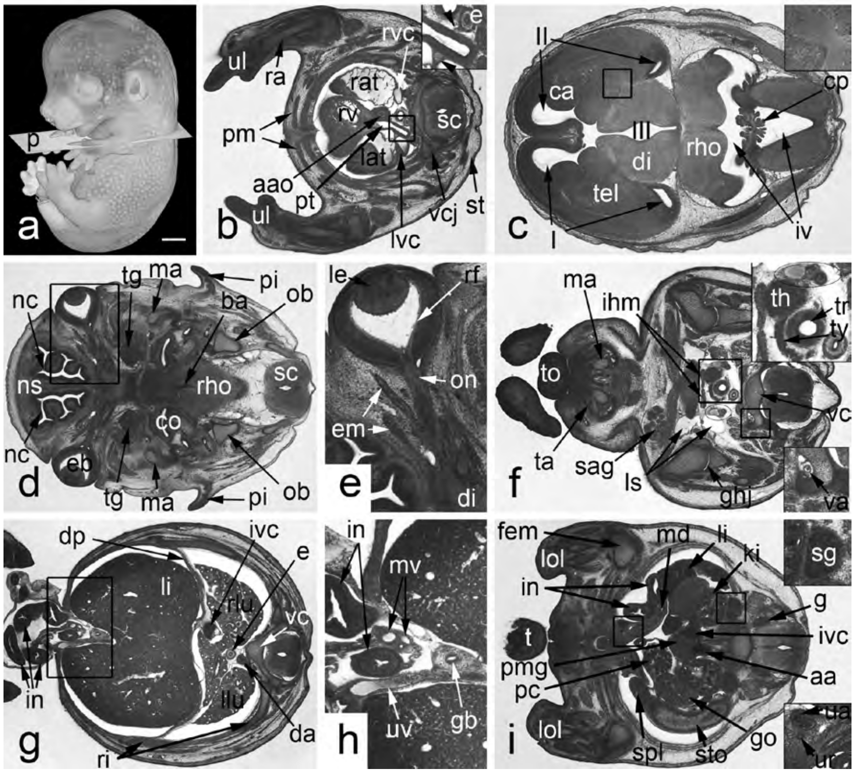

Supplementary Fig. 1.
Axial sections through HREM data of wild-type E14.5 mouse embryos. Selected important features are labelled. **a.** Volume rendered 3D model with one original section plane (p). Scalebar = 1mm. **b.** Section at the position indicated in a. Inlay shows the vagus nerves (arrows) lateral to the pulmonary trunk (pt) and ductus arteriosus (continuation of pt) respectively. **c.** Section through the neurocranium. The internal capsule is indicated by a box and magnified in the inlay. Note the choroid plexus (cp) of the 4th ventricle (iv). **d.** Section at the level of the eyes (eb). **e.** Magnification of the region boxed in d (orbit). Note the eye muscles (em) and the fibers forming the retina (rf) and the optic nerve (on). **f.** Section at the level of the glenohumeral joint (ghj). Note in the top inlay the cranial pole of the right thymus (th) and the isthmus of the thyroid gland (ty) ventral to the trachea (tr). Note in the bottom inlay the vertebral artery (va) in the foramen formed by the transverse process of the vertebra. An oval surrounds the common carotid artery, vagus nerve, and internal jugular vein. **g.** Section at the level of the gall bladder (gb). **h.** Magnification of the region boxed in g. Note the umbilical vein (uv), the mesenterial vessels (mv) and the physiologic umbilical hernia. **i.** Section at the level of the para-aortal bodies (pmg). Note in the top inlay the suprarenal gland (sg). Note in the bottom inlay the ureter (ur) and umbilical artery (ua).

[aa, abdominal aorta; aao, ascending aorta; ba, basilar artery; ca, caudate nucleus; co, cochlea; cp, choroid plexus; da, descending aorta; di, diencephalon; dp, diaphragm; e, esophagus; eb, eye ball; em, eye muscles; fem, femur; g, spinal ganglion; gb, gall bladder; ghj, glenohumeral joint; go, gonad; I, 1st ventricle; ihm, infrahyoid muscles; II, 2nd ventricle; III, 3rd ventricle; in, intestine; IV, 4th ventricle; ivc, inferior vena cava; ki, kidney; lat, left atrial appendix; li, liver; llu, left lung; lol, lower limb; ls, lymph sac; lvc, left vena cava superior; ma, mandible; mv, mesenterial vessel; nc, nasal cavity; ns, nasal septum; ob, occipital bone; on, optic nerve; p, pinna; pc, pancreas; pm, pectoralis muscle; pmg, para-aortal bodies; pt, pulmonary trunk; rat, right atrial appendix; rf, retinal fibres; rho, rhombencephalon; ri, rib; rlu, right lung; rv, right ventricle; rvc, right vena cava superior; sag, salivary gland; sc, spinal chord; sg, suprarenal gland; spl, spleen; st, subcutaneous tissue; sto, stomach; t, tail; ta, tooth primordium; tel, telencephalon; tg, trigeminal ganglion; th, thymus; to, tongue; tr, trachea; ty, thyroid gland; ua, umbilical artery; ul, upper limb; ur, ureter; uv, umbilical vein;va, vertebral artery; vc, body of vertebra; vcj, costovertebral joint].

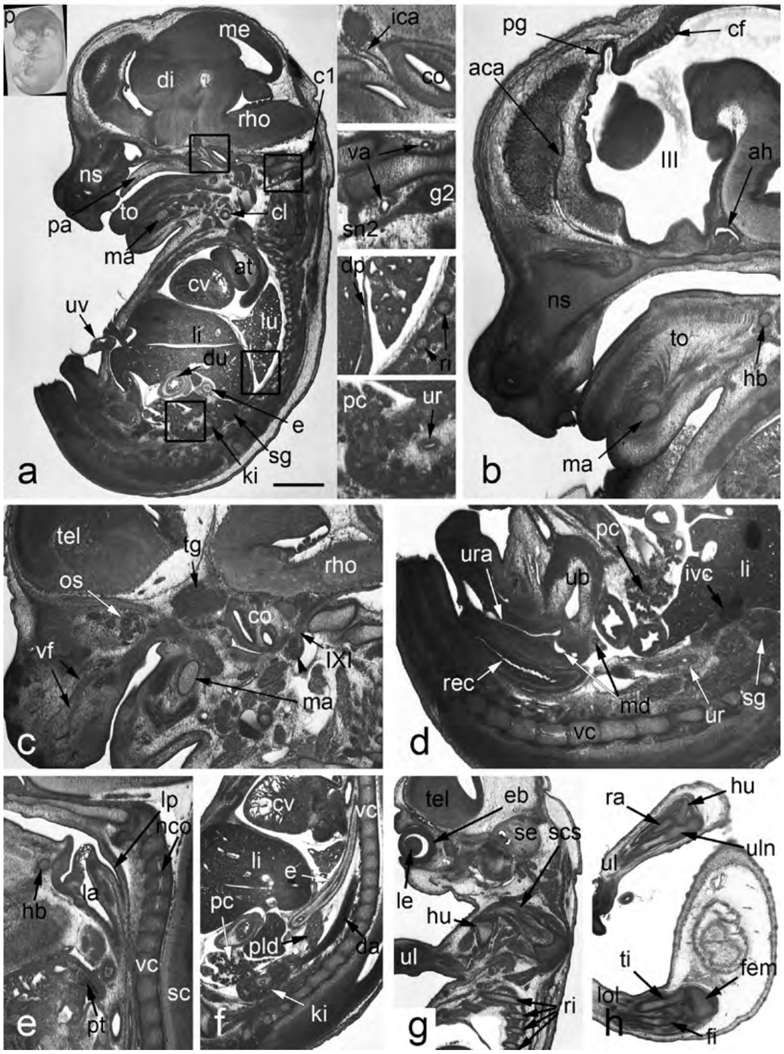

Supplementary Fig. 2.
Virtual sagittal re-sections through HREM data of wild-type E14.5 mouse embryos. Selected important features are labelled. **a.** Re-section through the embryo displayed in the inlay on the left. Note the section plane (p). Scalebar = 1 mm. Magnifications of the boxed areas are displayed as inlays on the right. Most cranial box corresponds to uppermost inlay. Note the internal carotid artery (ica), vertebral artery (va), spinal ganglion (g2) and spinal nerve (sn2) C2, diaphragm (dp), and ureter (ur) in the center of the pelvis of the kidney (ki). **b.** Anterior cerebral artery (aca) and pineal gland anlage (pg). Note the commissural fibers (cf), and adenohypohysis (ah). **c.** Nerve fibers innervating the vibrissae (vf) and cranial nerves IX, X, XI inside and below the jugular foramen. Note the ganglion of the vagus nerve (arrowhead), trigeminal ganglion (tg), and the eye muscles that surround the optic stalk (os). **d.** Urethra (ura), rectum (rec), and urinary bladder (ub). Note the distally enlarged mesonephric duct (md). **e.** Larynx (la). Note the laryngopharynx (lp) and the remnants of the notochord (nco) in the forming vertebral column. **f.** Lumbar part of diaphragm (pld). Note the openings for esophagus (e) and descending aorta (da). **g.** Eye (eb) and shoulder. Note the infraspinatus muscle below the spina scapulae (scs). **h.** Radius (ra) and ulna (ua), tibia (ti) and fibula (fi). Note the cubital joint and the knee joint.

[aca, anterior cerebral artery; ah, adenohypohysis; at, atrial appendix; cf, commissural fibers; cl, clavicle; co, cochlea; cv, cardiac ventricle; c1, atlas; da, descending aorta; di, diencephalon; dp, diaphragm; du duodenum; e, esophagus; eb, eye ball; fem, femur; fi, fibula; g2, spinal ganglion; hb, hyoid bone; hu, humerus; ica, internal carotid artery; III, 3rd ventricle; ivc, inferior vena cava; ki, kidney; la, larynx; li, liver; lol, lower limb; lp, laryngopharynx; lu, lung; ma, mandible; md, mesonephric duct; me, mesencephalon; nco, notochord; ns, nasal septum; os, optic stalk; pa, palatine; pc, pancreas; pg, pineal gland anlage; pld, Lumbar part of diaphragm; ra, radius; rec, rectum; rho, rhombencephalon; ri, rib; scs, spina scapulae; sg, suprarenal gland; sn2, spinal nerve; ti, tibia; tel, telencephalon; tg, trigeminal ganglion; to, tongue; ua, ulna; ub, urinary bladder; ul, upper limb; ur, ureter; ura, urethra; uv, umbilical vein; va, vertebral artery; vc; body of vertebra; vf, nerve fibers innervating the vibrissae].

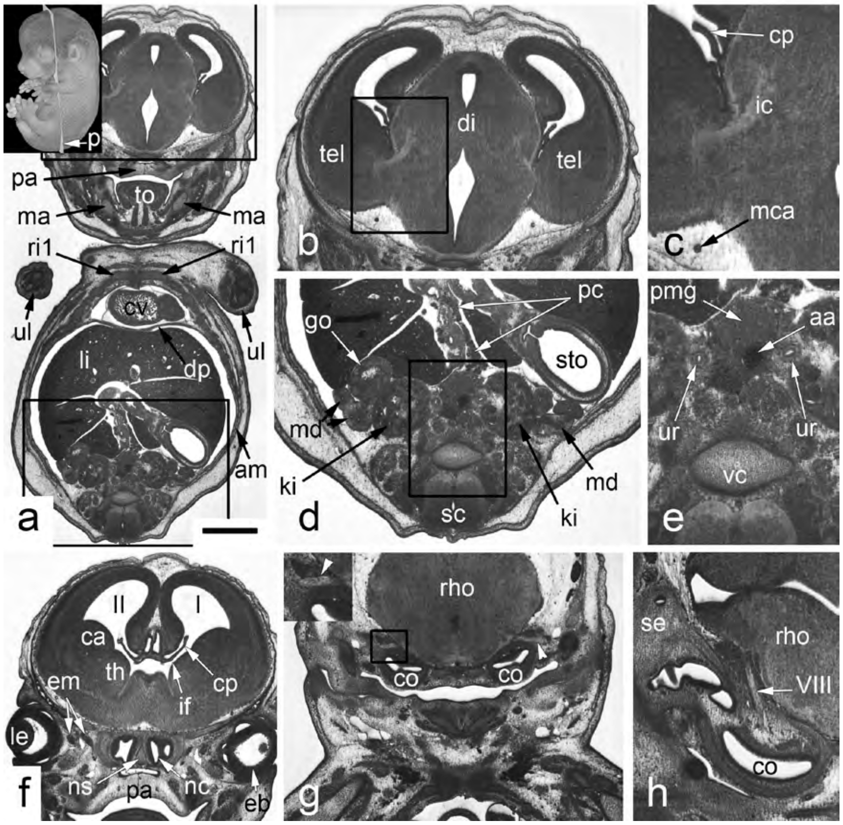

Supplementary Fig. 3.
Virtual coronal re-sections through HREM data of wild-type E14.5 mouse embryos. Selected important features are labelled. **a.** Re-section through the embryo displayed in the inlay on the left. Note the section plane (p). Scalebar = 1 mm. **b.** Magnification of the brain as boxed in a. **c.** Magnification of the region boxed in b. Note the fibers of the internal capsule (ic), choroid plexus (cp) of the 2nd ventricle, and middle cerebral artery (mca). **d.** Magnification of the pelvis as boxed in a. Note the pancreas (pa), kidneys (ki), and right gonad (go). **e.** Magnification of the region boxed in d. Note the para-aortic bodies (pmg), ureter (ur), and abdominal aorta (aa). **f.** brain, palatine (pa), and eye (eb). Note the forming caudate nucleus (ca) and thalamus (th). **g.** cranial nerve 7 (VII). Inlay shows a close up of the nerve (arrowhead), where it leaves the rhombencephalon (rho). **h.** Cranial nerve 8 (VIII). Pars cochlearis fibers enter the modiolus of the cochlea (co).

[aa, abdominal aorta; am, abdominal muscles; ca, caudate nucleus; co, cochlea; cp, choroid plexus; cv, cardiac ventricle; di, diencephalon; dp, diaphragm; eb, eye; em, eye muslces; go, gonad; I, 1st ventricle; ic, internal capsule; if, interventricular foramen; II, 2nd ventricle; ki, kidney; li, liver; le, lens; ma, mandible; mca, middle cerebral artery; md, mesonephric duct; nc, nasal cavity; pa, pancreas; pmg, para-aortic bodies; rho, rhombencephalon; ri1, 1st rib; sc, spinal chord; se, semicircular canals; sto, stomach; tel, telencephalon; th, thalamus; to, tongue; ul, upper limb; ur, ureter; vc, body of vertebra; VII, cranial nerve 7; VIII, cranial nerve 8].

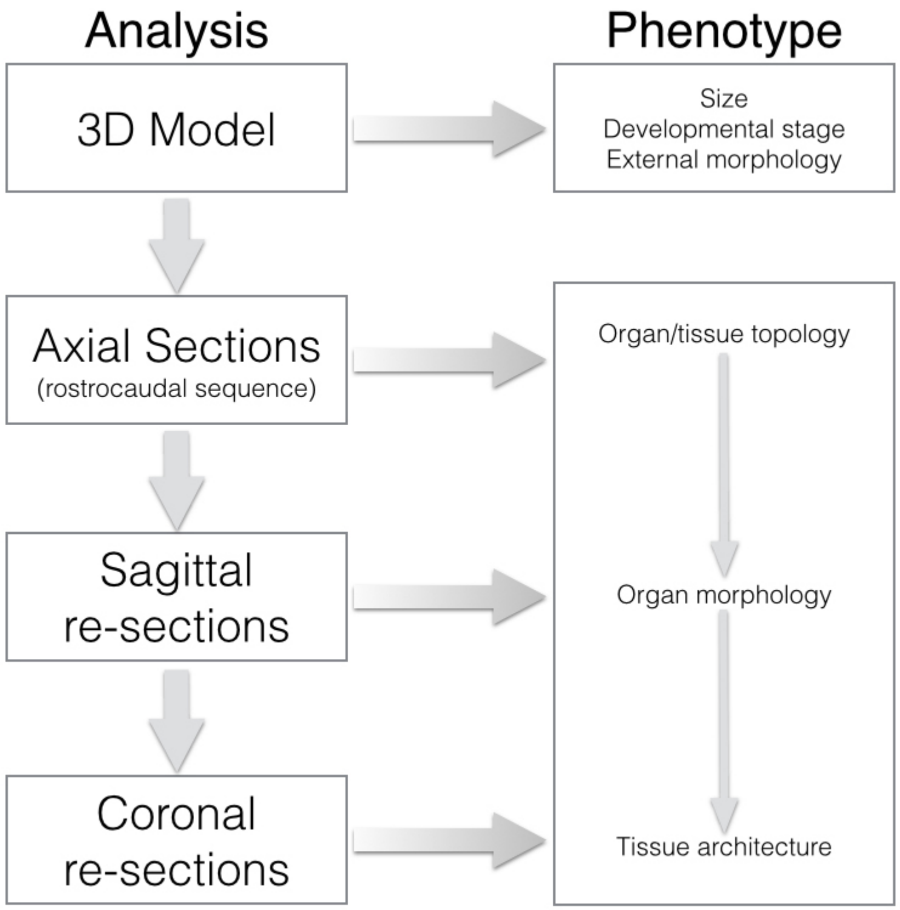

Supplementary Fig. 4.
Flow diagram of phenotyping protocol. Analysis proceeds from initial examination of embryo external appearance from 3D modelling to systematic examination of the HREM section image stack (in an overall rostrocaudal sequence). Organs and tissues to be assessed are listed in Table1. After completion of the axial sequence, Virtual resection data corresponding to sagittal and coronal section planes are similarly reviewed, following the checklists outlined (Table 1).

Supplementary Table 1.

Abnormalities diagnosed in the 34 different mouse strains analysed. Note that, according to their developmental stage, all embryos feature palatine cleft. We have therefore only used this term for embryos of developmental stage 23 (Theiler) with palatine plates lateral to their tongues. In addition, many embryos had remnants of the interventricular foramen, an observation that can be considered normal for the examined stages and is not therefore listed in this table.

Supplementary Table 1		
**Gene**	**Description**	**No.**
**Agpat3**	Abnormal eye cup morphology	1
	Shortening of cystic duct	1
**Akap9**	Subcutaneous edema	1
	Perimembranous ventricular septal defect	1
	Intramural bleeding in descending aorta	1
	Abnormal vertebral artery topology	1
**Aldh18a1**	Subcutaneous edema	2
	Eye ball inside head mesenchyme	3
	Absent lens vesicle	3
	Abnormal eye muscle development (morphology)	3
	Abnormal nasal cavity morphology	1
	Absent internal carotid artery segment	1
	Absent intrahepatic segment of vena cava inferior	1
**Amfr**	No phenotype	3
**Arid2**	Subcutaneous edema	3
	Exophthalmos	3
	Degeneration of brain tissue	1
	Abnormal heart position or orientation	3
	Perimembranous ventricular septal defect	3
	Double outlet heart right ventricle	3
	Double inlet heart left ventricle	2
	Muscular ventricular septal defect	3
	Transposition of great arteries	2
	Biventricular ambiguous atrioventricular connection	2
	Ostium primum atrial septal defect	2
	Retroesophageal right subclavian artery	2
	Retroesophageal left subclavian artery	1
	Right sided aortic arch	1
	Vascular ring around esophagus	1
	Abnormal vertebral artery segments	

	Blood in lymph sac	1
	Abnormal topology of vagus nerve	3
	Liver hypoplasia	1
	Liver cysts	1
	Abnormal liver morphology	1
	Absence of sternal extremitas of clavicle	1
**Arid4a**	No phenotype	3
**Asxl1**	Holoprosencephaly	2
	Absent internal capsule	3
	Abnormal 3rd ventricle morphology	1
	Absent olfactory bulb	1
	Narrow eye opening (or absent)	2
	Abnormal optic stalk morphology	3
	Abnormal optic cup morphology	3
	Anophthalmia	1
	Absent lens vesicle	3
	Abnormal eye muscle development	1
	Abnormal adenophypophysis morphology	2
	Multiple craniopharyngeal ducts	3
	Abnormal nasal septum morphology	1
	Abnormal nasal cavity morphology	1
	Ostium primum atrial septal defect	1
	Biventricular ambiguous atrioventricular connection	1
	Abnormal anterior cerebral artery	2
	Abnormal vertebral artery topology	1
	Absent internal carotid artery segment	1
**Cbx1**	Subcutaneous edema	3
	Persisting craniopharyngeal duct	3
	Abnormal aortic valve cusp morphology	1
	Abnormal pulmonary valve cusp morphology	1
	Anastomosis of internal carotid and basilar artery	2
	Abnormal basilar artery segments	2
	Abnormal vertebral artery topology	1
	Aneurysm of ocular artery	
	Abnormal vitelline vein connection	1
	Short cystic duct	1
	Abnormal small & large intestine placement	1

	Herniated liver	1
	Thyroid hypoplasia	2
**Cenpj**	Subcutaneous edema	1
	Persistent right umbilical vein (small)	1
	Abnormal small & large intestine placement	1
	Blood in lymph sac	1
**Cntfr**	No phenotype	3
**Csrp2bp**	Embryonic growth retardation	1
	Right sided aortic arch	2
	Retroesophageal left subclavian artery	2
	Narrow eye opening	2
	Eye ball formed inside head mesenchyme	2
	Abnormal optic cup morphology	1
	Abnormal optic stalk morphology	2
	Absent lens vesicle	2
	Vacuolated lens	1
	Abnormal eye muscle development (morphology)	2
	Abnormal mouth morphology	1
	Abnormal nasopharynx morphology	1
	Absent tongue	1
	Right sided aortic arch	2
	Retroesophageal left subclavian artery	2
	Abnormal external carotid artery origin	1
	Blood in lymph sac	1
	Absent tooth primordium	1
**Dot1l**	Persisting craniopharyngeal duct	2
	Palatine cleft	3
	Perimembranous ventricular septal defect	3
	Thin myocardium compact layer	2
	Blood in lymph sac	2
	Abnormal large & small intestine placement	3
	Abnormal caecum topology	1
	Caecum inside body	1
	Fusion of vertebral bodies	3
	Occipital vertebra	3
	Fusion of vertebral arches	2

**Dip2b**	Subcutaneous edema	1
	Absence of hypoglossal nerve	2
	Abnormal topology of hypoglossal nerve	1
	Absent hypoglossal canal	3
	Perimembranous ventricular septal defect	1
	Overriding aortic valve	1
	Abnormal topology of vagus nerve	1
	Abnormal vertebral artery topology	1
	Blood in lymph sac	1
**Dscc1**	Abnormal heart position or orientation	1
	Perimembranous ventricular septal defect	1
	Double outlet heart right ventricle	1
	Muscular ventricular septal defect	1
	Persisting right dorsal aorta	1
	Abnormal origin of external carotid artery	1
	Abnormal vertebral artery topology	1
	Absent ductus venosus	1
	Dual inferior vena cava	1
	Liver veins drain into left vena cava superior	1
	Liver cysts	2
	Abnormal vagus nerve topology	1
	Herniated liver	1
	Lumbar rib	3
**Ehd1**	Abnormal lens morphology	2
	Retrolental bleeding	1
	Absent lens vesicle	1
	Abnormal optic cup morphology	2
	Perimembranous ventricular septal defect	1
	Overriding aortic valve	1
	Muscular ventricular septal defect	1
	Abnormal small & large intestine placement	3
	Abnormal kidney morphology	2
	Bifid ureter	1
**Fam134c**	Thin myocardium compact layer	1
	Intestinal/bowel diverticulum	1
	Abnormal perirectal tissue morphology	1

**Gap43**	No phenotype	3
**Grin1**	Subcutaneous edema	1
	Additional intracranial anastomosis of vertebral arteries	1
	Persisting right umbilical vein (small)	1
	Duplication of cystic duct	1
**Ikbkb**	Embryonic growth retardation	2
	Decreased embryo size	1
	Abnormal eye distance/position	1
**Jarid2**	Embryonic growth retardation	1
	Subcutaneous edema	2
	Mediastinal edema	1
	Perimembranous ventricular septal defect	1
	Overriding aortic valve	1
	Abnormal vertebral artery origin	1
	Blood in lymph sac	1
**Lmnb2**	Abnormal large intestine placement	1
**Ltbp1**	Subcutaneous edema	3
	Small superior cervical ganglion	1
	Perimembranous ventricular septal defect	3
	Double outlet heart right ventricle	3
	Abnormal heart atrium morphology	1
	Dual inferior vena cava	2
	Persistent right umbilical vein	1
	Thyroid lobe hypoplasia	1
**Mbtd1**	Embryonic growth retardation	1
	Abnormal eye muscle development	1
	Perimembranous ventricular septal defect	2
	Muscular ventricular septal defect	1
	Double outlet heart right ventricle	2
	Thin myocardium compact layer	2
	Transposition of great arteries	1
	Interrupted aortic arch, type B	1
	Persisting right dorsal aorta (Kommerell diverticle)	1

	Right sided aortic arch	1
	Retroesophageal left subclavian artery	1
	Abnormal kidney morphology	1
	Single kidney	2
**Mks1**	Embryonic growth retardation	2
	Subcutanous edema	2
	Thoracoschisis	1
	Podydactyly	3
	Abnormal maxillary prominence morphology	2
	Abdominal situs inversus	1
	Abdominarl suits ambiguus	1
	Situs ambiguus	1
	Fibroma in brain tissue	2
	Hemangioma in brain	1
	Abnormal adenohypophysis morphology (2 of 3 absent)	3
	Abnormal neurohypophysis morphology (2 of 3 absent)	3
	Multiple craniopharyngeal ducts	1
	Persisting craniopharyngeal duct	1
	Abnormal pineal gland morphology	2
	Anophthalmia	3
	Abnormal optical stalk morphology	3
	Absence of single eye muscles	1
	Absent hypoglossus nerve	1
	Malformed olfactory bulb	3
	Abnormal heart position or orientation	1
	Perimembranous ventricular septal defect	3
	Double outlet heart right ventricle	3
	Double inlet heart left ventricle	1
	Transposition of great arteries	3
	Abnormal semilunar valve cusp morphology	1
	Muscular ventricular septal defect	3
	Common atrium	2
	Left atrium isomerism	1
	Misalignment of atrial appendices	1
	Left atrial appendix part of right atrium	1
	Absent pulmonary valve cusps	1
	Single lung vein	1
	Two lung veins	1
	Lung veins drain into left vena cava superior	1

	Double lumen aortic arch	1
	Persisting right sided aorta	1
	Right sided aortic arch	1
	Vascular ring around esophagus	2
	Abnormal vertebral artery origin	2
	Left sided brachiocephalic trunk	1
	Absent left lung	1
	Left pulmonary isomerism	2
	Tracheoesophageal fistula	1
	Abnormal basilar artery segments	3
	Anastomosis of internal carotid and basilar arteries	2
	Dual inferior vena cava	3
	Symmetric hemiazygos/azygos veins	3
	Symmetric liver veins	1
	Symmetric dual inferior vena cava	1
	Absent intrahepatic segment of vena cava inferior	2
	Abnormal umbilical vein topology	1
	Blood in lymph sac	2
	Right sided stomach	1
	Abnormal small & large intestine placement	2
	Hemangioma in liver	1
	Hemangioma in pericardium	1
	Fibroma in adrenal gland	1
	Fibroma in paraaortic bodies	1
	Symmetric appearance of liver	1
	Abnormal vagus nerves topology	1
	Additional canal in occipital bone	2
	Absent hypoglossal canal	1
	Abnormal basisphenoid bone	1
	Rib fusion	1
	Abnormal cervical rib	1
	Absence of sternal extremitas of clavicle	1
**Mysm1**	Embryonic growth retardation	2
	Growth retardation of single organs	1
	Abnormal maxillary prominence	1
	Absent nasal capsule	1
	Absent hard palate	1
	Abnormal brain morphology	3
	Enlarged 3rd ventricle	1

	Delayed brain development	1
	Absent olfactory bulb	1
	Eye ball formed inside head mesenchyme	1
	Abnormal optic cup morphology	3
	Abnormal optic stalk morphology	2
	Absent temporal retina	1
	Absent lens	2
	Abnormal eye muscle development	2
	Multiple craniopharyngeal ducts	1
	Perimembranous ventricular septal defect	2
	Double outlet right ventricle	1
	Overriding aortic valve	1
	Muscular ventricular septal defect	1
	Abnormal cerebral anterior artery	1
	Anal atresia	1
**Nf1**	Embryonic growth retardation (two mazerated)	2
	Abnormal brain morphology	1
	Degeneration of brain tissue	1
	Abnormal heart position or orientation	2
	Abnormal heart atrium morphology	3
	Perimembranous ventricular septal defect	3
	Double outlet heart right ventricle	3
	Transposition of great arteries	1
	Persistent truncus arteriosus	2
	Interrupted aortic arch, typeB	2
	Retroesophageal right subclavian artery	2
	Abnormal vertebral artery topology	3
	Abnormal basilar artery segments	2
	Opening in diaphragm	1
	Abnormal adrenal gland morphology	1
**Pds5b**	Growth retardation of single organs	3
	Subcutaneous edema	3
	Small superior cervical ganglion	3
	Perimembranous ventricular septal defect	3
	Overriding aortic valve	3
	Muscular ventricular septal defect	2
	Thin myocardium compact layer	1
	Absent internal carotid artery segment	2

	Abnormal internal carotid artery topology	2
	Abnormal vertebral artery topology	2
	Abnormal vitelline vein connection	2
	Abnormal large and small intestine topology	1
	Liver hypoplasia	1
	Absence of hypoglossal nerve	1
**Prkab1**	Dual ductus venosus	1
	Absent superior cervical ganglion	1
	Absent labyrinthine artery	1
	Hemangioma in brain	1
**Prmt3**	Embryonic growth retardation	1
	Enlarged lateral ventricles	1
	Abnormal pineal gland morphology	3
	Abnormal olfactory bulb position	1
	Abnormal optic cup morphology	1
	Duplication of osseous nasal septum	1
	Perimembranous ventricular septal defect	1
	Overriding aortic valve	1
	Abnormal vertebral artery segments	1
	Abnormal vitelline vein connection	1
**Psat1**	Embryonic growth retardation	3
	Subcutaneous edema	3
	Mediastinal edema	3
	Abnormal maxillary prominence	3
	Abnormal outer ear morphology	3
	Holoprosencephaly	3
	Enlargement of supratentorial ventricles	2
	Delayed brain development	1
	Absent internal capsule	3
	Abnormal pineal gland morphology (absent)	2
	Abnormal optic stalk morphology	3
	Absent lens vesicle	2
	Eye ball inside head mesenchyme	3
	Abnormal eye muscle development (absent)	2
	Absent hard palate	3
	Abnormal external auditory canal morphology	3
	Cystic structures in head	3

	Absent nasal capsule	1
	Abnormal nasal cavity morphology	2
	Abnormal vomeronasal organ morphology	3
	Absent olfactory nerve	3
	Abnormal mandible morphology	3
	Absent tooth primordium	3
	Abnormal clavicle morphology	2
	Abnormal basisphenoid bone morphology	3
	Abnormal tongue muscle morphology	3
	Perimembranous ventricular septal defect	3
	Double outlet heart right ventricle	3
	Persistent truncus arteriosus	2
	Aortopulmonary window	1
	Retroesophageal right subclavian artery	1
	Abnormal pulmonary artery origin	1
	Abnormal vertebral artery topology	3
	Abnormal valve of ductus venosus topology	2
	Absent intrahepatic segment of vena cava inferior	1
	Abnormal vagus nerve topology	2
	Absent lobe of thyroid gland	1
	Abnormal thymus topology	1
	Opening in diaphragm	1
	Absent spleen	1
	Spleen hypoplasia	2
	Pancreas hypoplasia	3
	Abnormal kidney morphology	3
**Rhot1**	Abnormal vertebral artery topology	2
	Blood in lymph sac	1
**Suv420h1**	Subcutaneous edema	1
	Thoracoschisis	1
	Ectopia cordis	1
	Abnormal mandibular prominence	1
	Perimembranous ventricular septal defect	1
	Double outlet heart right ventricle	1
	Abnormal heart atrium morphology	1
	Abnormal vertebral artery topology	2
	Abnormal umbilical vein topology	1
	Abnormal mandible morphology	1

	Absent tooth primordium	1
	Absent tongue muscles	1
	Absent salivary gland	1
	Abnormal vagus nerve topology	1
	Herniated liver	1
	Abnormal small & large intestine topology	1
**Supv3l1**	No phenotype	3
**Zc3hc1**	Embryonic growth retardation	1
	Abnormal optic cup morphology	1
	Abnormal lens morphology	1
	Persisting dorsal ophthalmic artery	1
	Abnormal vagus nerve topology	1
	Abnormal thymus topology	1
	Rib bifurcation	1
	Intestinal/bowel diverticulum	1

Supplementary Table 2.
Abnormalities diagnosed in embryos from 34 mutant mouse lines at 14.5 dpc, after analysis by HREM. Phenotype terminology is according to systematic MP lists (http://www.ebi.ac.uk/ontology-lookup/browse.do?ontName=MP). Note the brief anatomical description, which specifies or extends the abnormality if MP terms are not sufficiently descriptive. Column L (lethality): estimate of severity of defects according to the categories Sc1-Sc5 defined in the text. Column Q: estimate of data quality and resolution at which the given abnormalities can be diagnosed reproducibly (m: μMRI data quality and lower; i: intermediate level of data quality; h: histological or HREM level of data quality. Note that diagnosis of certain abnormalities depends on their severity, reflected in estimates i/h, m/h, and m/I (shown in parentheses).

Download Table S2
